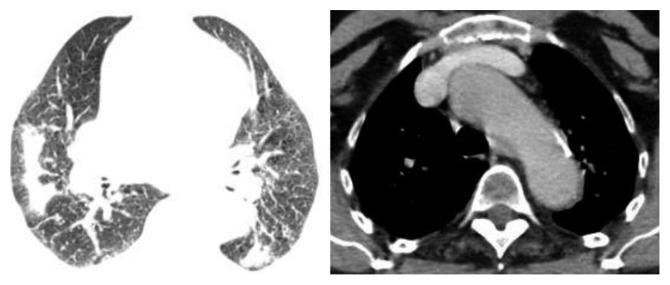# Pulmonary Sarcoidosis following Etanercept Treatment for Ankylosing Spondylitis: A Case Report and Review of the Literature

**DOI:** 10.1155/2018/9867248

**Published:** 2018-01-23

**Authors:** A. Majjad, A. Bezza, A. Biyi, M. R. El Ochi, A. El Maghraoui

**Affiliations:** ^1^Rheumatology Department, Mohammed V Military Academic Hospital, Faculty of Medicine and Pharmacy, Mohammed V University, Rabat, Morocco; ^2^Nuclear Medicine Department, Mohammed V Military Academic Hospital, Faculty of Medicine and Pharmacy, Mohammed V University, Rabat, Morocco; ^3^Anatomic Pathology Department, Mohammed V Military Academic Hospital, Faculty of Medicine and Pharmacy, Mohammed V University, Rabat, Morocco

## Abstract

Antitumor necrosis factor therapies have revolutionized the treatment of some inflammatory diseases. However, the use of these agents is associated with the development of many paradoxical autoimmune diseases. Less well-recognized is the association with sarcoidosis. We report a 55-year-old female with long-standing ankylosing spondylitis who developed persistent dry cough and dyspnea while receiving etanercept therapy. High-resolution computed tomography scanning showed mediastinal lymphadenopathy and multiple nodules in both lung fields developed two months after the administration of etanercept. Lymph node biopsy was not practicable. Histopathological examination of minor salivary gland biopsy revealed noncaseating granulomata, and the serum angiotensin-converting enzyme was very elevated. All infectious studies were negative. Etanercept was discontinued plus a course of corticosteroids with a clinical improvement, and a follow-up high-resolution computed tomography scanning 4 months later showed evident regression of mediastinal lymph nodes and pulmonary nodules. Potential pathogenic mechanisms of this paradoxical effect of tumor necrosis factor-alpha blocking agents are discussed.

## 1. Background

Sarcoidosis is a rare granulomatous disease mainly affecting the lungs and lymph nodes. Its etiology remains unknown [1]. However, there are a small number of literatures possible that involve tumor necrosis factor-alpha blocking agents (anti-TNF-*α*), especially etanercept, in the development of pulmonary sarcoidosis [2]. Our case report supports possible relationship between etanercept therapy and the development of sarcoid-like granulomatosis.

## 2. Case Report

**Figure 1:**
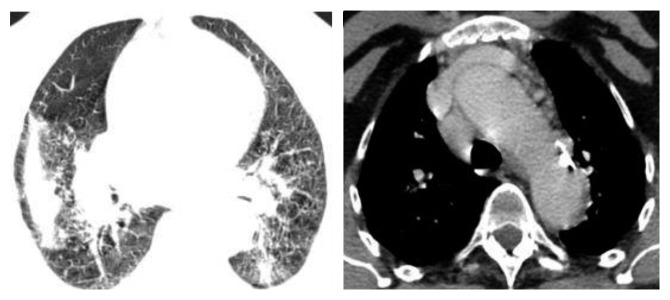


**Figure 2:**
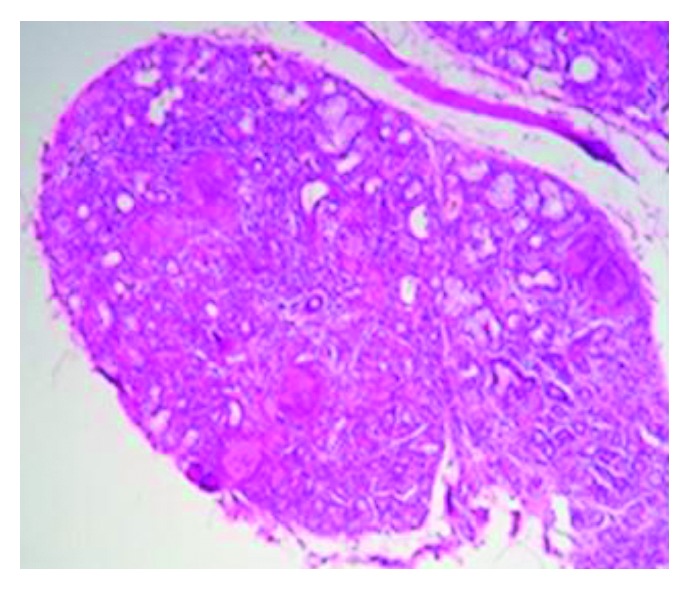


A 66-year-old female patient without medical history or professional exposures was diagnosed with ankylosing spondylitis 12 years ago. She had formerly responded to nonsteroidal inflammatory drugs. In 2006, she started treatment with infliximab, due to poor therapeutic response to various NSAIDs. After eight years of remission, the patient had repeated flares of ankylosing spondylitis symptoms with increased inflammatory back pain. In August 2015, due to persistent clinical activity with increased acute-phase reactant levels (C-reactive protein level of 16 mg/l), the treatment with etanercept at 25 mg twice weekly was started. Following national guidelines to exclude latent tuberculosis, mycobacterium tuberculosis research in sputum and interferon-gamma test were negative and a chest radiograph was normal. Two months after etanercept therapy onset, the patient developed persistent dry cough and dyspnea without fever or night sweating. Physical examination was unremarkable apart from tachypnea and severe spinal stiffness. High-resolution computed tomography (HRCT) scanning showed mediastinal lymphadenopathy and multiple nodules in both lung fields (Figure 1). Echocardiography was normal. The blood analysis demonstrated ESR 35 mm/h, CRP 14 mg/l, and normal renal and hepatic function tests. Sputum culture and polymerase chain reaction and interferon-gamma release assays were negative. Viral serology (human immunodeficiency virus, hepatitis B virus, and hepatitis C virus) and syphilis serology were negative. Serum angiotensin-converting enzyme was very elevated at 147 UI/ml (reference range 8–52 UI/ml). Calcemia and calciuria tests were normal. The pulmonary function test showed restrictive ventilatory alteration. It was not possible to perform neither bronchoscopy nor thoracoscopy because of respiratory failure and severe ankylosis of the cervical spine. Histopathological examination of minor salivary gland biopsy revealed a chronic granulomatous sialadenitis without caseous necrosis (Figure 2). Diseases showing similar histologic changes were excluded, and a diagnosis of sarcoidosis was made. Etanercept was discontinued plus a course of corticosteroids with 40 mg/day of prednisone for two months. An evident improvement of cough and dyspnea was seen. Follow-up HRCT 4 months later showed regression of mediastinal lymph nodes and pulmonary nodules (Figure 3).

## 3. Discussion

Sarcoidosis is a multisystem disorder characterized by the accumulation of lymphocytes, mononuclear phagocytes, and noncaseating granulomas in involved tissues. Etiology of sarcoidosis is still not identified. The role of viral, bacterial, and parasitic infections is not clearly determined. The role of some drugs and environmental exposure are still considered [3]. Due to unknown causes, active macrophages and lymphocytes are accumulated in this or that organ and produce increased number of interleukins, TNF-*α*. The TNF-*α* is the key cytokine that participates in the formation of granulomas in sarcoidosis [3, 4].

At present, there are five anti-TNF-*α* agents currently available: four monoclonal antibodies (infliximab, adalimumab, golimumab, and certolizumab) and a soluble TNF-*α* receptor fusion protein (etanercept) [5]. Anti-TNF-*α* agents, especially infliximab and sometimes adalimumab, are effective in refractory sarcoidosis [6, 7]. However, there have been a few cases of sarcoidosis occurring during anti-TNF-*α* therapy. In a recent review of literature published on January 2016, SimJae Kyeom et al. found 59 cases of sarcoidosis occurring during anti-TNF-*α* therapy in the medical literature. Thirty-seven (61.6%) occurred after etanercept, 13 (21.6%) after adalimumab, and 9 (15%) after infliximab. In this study, twenty-eight had rheumatoid arthritis. The lung and/or lymph node were the most commonly affected organs (64%), followed by the skin (37%) and the eye (15%). Prognosis seems to be favorable since 52 of 59 cases displayed a partial or complete resolution after anti-TNF-*α* withdrawal with or without steroids [2].

The pathogenesis of sarcoidosis induced by anti-TNF-*α* remains unclear. However, some potential hypotheses have been proposed. Mainly followed hypothesis is that sarcoidosis is of an infectious origin favored by an immunosuppressant effect of anti-TNF-*α*, especially with some microorganisms that are implicated in the development of sarcoidosis (*Propionibacterium acnes* and *Propionibacterium granulosum*) [8]. Another hypothesis is that the disease is an immunological disorder, with an imbalance in Th17 lymphocytes and Treg lymphocytes, favoring Th17 and formation of granulomas via the increased expression of IL17 and TNF-*α*. Currently, there are many reports supporting this latter hypothesis [9–11]. Indeed, more than 60% of the reported cases occurred with etanercept. There are two hypotheses that may explain this greater association: on one hand, the difference in pharmacological proprieties between the synthesized anti-TNF-*α* receptor and monoclonal antibodies: the antibodies neutralize both soluble and membrane forms of TNF-*α*, whereas etanercept neutralizes just the soluble form. This partial inhibition permits the redistribution of TNF-*α* in areas of elevated concentration such as the lungs and lymph nodes [11]. On the other hand, several studies suggest that monoclonal antibodies, in contrast to etanercept, inhibit the expression of IL17 and INF-gamma, cytokines strongly involved in granuloma formation [12]. Taking into account that it is not the only case of sarcoidosis developed with the anti-TNF therapy, the pathogenesis of granulomas under the effect of the therapy needs to be studied. Besides, it is possible that similar complications of anti-TNF therapy may be developed in carriers of HLA-A1, B8, and B13 antigens as it is known that those antigens are met more frequently in sarcoidosis patients compared to the general population [13]. In summary, it is important for clinicians to be aware of this rare complication of anti-TNF therapy. Sarcoidosis granulomas may be formed not only in the intrathoracic lymphatic nodes, lungs, and salivary glands but in other organs too (liver, spleen, eyes, heart, skin, etc.). If the biopsy of the affected organ is difficult, minor salivary gland biopsy may be an excellent alternative and help in diagnosis [11, 13].

## 4. Conclusion

Pulmonary sarcoidosis is an uncommon effect of anti-TNF therapy. The pathogenic mechanism is unclear. Minor salivary gland biopsy may help in diagnosis. The management requires discontinuing anti-TNF plus a course of steroids.

## Figures and Tables

**Figure 3:**